# The Accuracy of Sonographically Estimated Fetal Weight and Prediction of Small for Gestational Age in Twin Pregnancy—Comparison of the First and Second Twins

**DOI:** 10.3390/jcm12093307

**Published:** 2023-05-06

**Authors:** Moran Gawie-Rotman, Shoval Menashe, Noa Haggiag, Alon Shrim, Mordechai Hallak, Rinat Gabbay-Benziv

**Affiliations:** 1 Obstetrics and Gynecology Division, Hillel Yaffe Medical Center, Hadera 3846201, Israel; moran.gawie7@gmail.com (M.G.-R.);; 2 The Ruth and Bruce Rappaport Faculty of Medicine, Technion-Israel Institute of Technology, Haifa 3200003, Israel

**Keywords:** twin pregnancy, small for gestational age, estimated fetal weight, accuracy of birthweight

## Abstract

Accurate sonographic estimation of fetal weight is essential for every pregnancy, especially in twin gestation. We conducted a retrospective analysis of the sonographically estimated fetal weight (sEFW) of all twin gestations performed within 14 days of delivery in a single center that aimed to evaluate the accuracy of sEFW in predicting neonatal weight and small for gestational age (SGA) by comparing the first fetus to the second. A total of 190 twin gestations were evaluated for the study. There was no statistically significant difference in the sEFW between the first and the second twins, but the second twin had a statistically significant lower birth weight (2434 vs. 2351 g, *p* = 0.028). No difference was found in median absolute systematic error (*p* = 0.450), random error, or sEFW evaluations that were within 10% of the birth weight between the fetuses (65.3% vs. 67.9%, *p* = 0.587). Reliability analysis demonstrated an excellent correlation between the sEFW and the birth weight for both twins; however, the Euclidean distance was slightly higher for the first twin (12.21%). For SGA prediction, overall, there was a low sensitivity and a high specificity for all fetuses, with almost no difference between the first and second twins. We found that sEFW overestimated the birth weight for the second twin, with almost no other difference in accuracy measures or SGA prediction.

## 1. Introduction

Twin pregnancies are associated with a high incidence of pregnancy complications. One of the most prevalent risks is preterm delivery, which accounts for most of the increased perinatal morbidity and mortality. Additionally, higher rates of fetal growth abnormalities and congenital anomalies contribute to adverse outcomes in twin pregnancies [1].

It has been suggested that neonatal morbidity and mortality tend to be higher for the second-born twin (as compared to the first-born). In a systematic review of observational studies, overall neonatal morbidity, defined as pH < 7.0, Apgar score < 7 at 5 min, or any neonatal birth trauma, was 3.0 and 4.6 percent, respectively (OR 0.53, 95% CI 0.39–0.70), and overall neonatal mortality, defined as death within 28 days, of the first and second twins was 0.3 and 0.6 percent, respectively (OR 0.55, 95% CI 0.38–0.81). The increased risk of adverse neonatal outcomes in the second-born twin was most likely related to a lower birth weight, a higher frequency of malpresentation, cord prolapse, placental abruption, and the need for obstetric maneuvers at delivery [2].

In twin gestation, monitoring the fetus’s growth is of utmost importance. According to the International Society of Ultrasound in Obstetrics and Gynecology (ISOUG) guidelines, sonographic evaluation of fetal growth is recommended every four weeks for uncomplicated bichorionic twins and every two weeks for uncomplicated monochorionic twins [3]. Accurate follow-up is imperative for the early detection of peripartum placental insufficiency, ultimately allowing the healthcare provider to prepare for complications that may arise during childbirth. Unfortunately, sonographic estimation of fetal weight (sEFW) has been proven to be less accurate in twins than in singleton pregnancies [4]. 

For a singleton pregnancy, the mean error between the sEFW and the neonate’s birth weight is about 10–20% [5,6,7]. The degree of accuracy depends on the examiner and on the fetal and maternal parameters, such as fetal presentation, gestational age, amniotic fluid volume, and the level of maternal obesity [8,9]. For twin pregnancies, despite the high incidence of growth abnormalities, only a few studies in the literature have evaluated the accuracy of sEFW. Furthermore, these studies have presented conflicting results [10,11,12,13].

This study aims to evaluate the accuracy of ultrasound in the prediction of neonatal birth weight with an emphasis on comparing the first fetus (closer to the cervix) with the second fetus. Moreover, we calculated the accuracy measurements for the determination of small for gestational age (SGA), defined as sEFW under the tenth percentiles for the two fetuses, and compared them.

## 2. Materials and Methods

### 2.1. Population

This was a retrospective cohort analysis of women carrying twin gestations who delivered in a single, tertiary, university-affiliated medical center. All twin pregnancies delivered between September 2011 and August 2021, in which sonographic fetal biometry estimation was performed within 14 days before deliveries, were analyzed. The study was approved by the local Institutional Review Board committee (HYMC-0048-22). Due to the retrospective nature of the study, informed consent was waived. Inclusion criteria included all live twin births who had a sonographic evaluation within 14 days before delivery. Cases with any known chromosomal abnormalities or major malformations were excluded. In addition, we excluded women without available full documentation of all biometric measurements (biparietal diameter (BPD), head circumference (HC), abdominal circumference (AC), and femur length (FL), as well as women who were in active labor or with ruptured membranes at the time of the sonographic assessment. Cases with unclear chorionicity or suspected growth abnormalities were not excluded.

### 2.2. Data

Data were retrieved from the comprehensive computerized database of sonographic examinations and compared to the perinatal database. Matching was verified by comparing the date of the last menstrual period to avoid mixing data from two different pregnancies of the same woman. The gestational age at the time of the sonographic evaluation was calculated by the last menstrual period or by first-trimester ultrasound if a discrepancy exceeding six days between them was present. Antenatal data, including the gestational age at delivery and the actual birth weights, were obtained from the perinatal database. Small for gestational age was defined as neonates under the 10th percentile using twins, gestational age, and gender-specific customized curves, constructed based on our population [14]. The sonographic sEFW was calculated for every twin using the Hadlock formula: (EFW(hadlock_4) = 10^(1.3596+0.0064×Q2+0.0424×R2+0.174×S2+0.00061×P2×R2−0.00386×R2×S2)^).

### 2.3. Measurements

By convention, fetal sonographic evaluations included all standard fetal biometry measurements (AC, FL, BPD, and HC) according to ISUOG guidelines [3], presenting part, placental location, and amniotic fluid estimation for every twin, measured by the largest vertical pocket. All examinations were performed trans-abdominally using a high-quality ultrasound system, GE Voluson E6, Voluson E8, or Voluson E10 (GE Medical Systems, Zipf, Austria), by physicians who are ultrasound specialists or by experienced ultrasound technicians. Twin A (the 1st twin) was defined as the fetus closer to the cervix. The BPD was measured from the proximal echo of the fetal skull to the proximal edge of the deep border (outer–inner) at the level of the cavum septum pellucidum. The HC was measured as an ellipse around the perimeter of the fetal skull at the same level [15]. The AC was measured in the transverse plane of the fetal abdomen at the level of the umbilical vein in the anterior third and the stomach bubble in the same plane; measurements were taken around the perimeter [16]. The FL was measured in a view in which the full femoral diaphysis was seen and was taken from one end of the diaphysis to the other, not including the distal femoral epiphysis [17]. After birth, neonatal birth weight and anthropometric data were immediately documented. Neonate A (1st neonate) was defined as the first twin delivered. 

### 2.4. Accuracy and SGA Evaluation

For every twin fetus, the sEFW was evaluated and compared to the neonatal birth weight. Accuracy was evaluated for every twin and compared between the 1st (closer to the cervix) and 2nd fetus. Measures of accuracy included the systematic error (calculated as the absolute [sEFW − birth weight]/birth weight × 100, reflecting the systematic deviation of the sEFW from the birth weight, expressed as a percentage of the birth weight); the random error (the standard deviation of the systematic error), reflecting the random component of prediction error; and the proportion of estimates within 10% of the birth weight. To further compare the accuracy of EFW between the 1st and 2nd twins, we utilized the Euclidean distance (=square root of [systematic error^2^ + random error^2^]), representing the geometric average of the systematic and random errors. 

Next, to evaluate the sEFW prediction of SGA at birth for every twin, we compared the sEFW and the neonatal birth weight with the 10th percentile for the exact gestational age. Accuracy was then evaluated using the following measures: sensitivity, specificity, positive predictive value (PPV), negative predictive value (NPV), positive likelihood ratio (+LR, defined as sensitivity/(1 − specificity)), and negative likelihood ratio (−LR, defined as (1 − sensitivity)/specificity). Overall accuracy was defined as (true negative + true positive cases)/all cases.

### 2.5. Statistical Analysis

Statistical analysis was performed using SPSS version 28.0 software (SPSS, Inc., Chicago, IL, USA). *p* < 0.05 was considered significant. Categorical data were analyzed using Fisher’s exact test, and continuous variables were compared using the Mann–Whitney–Wilcoxon test as appropriate. Reliability analysis was used to calculate the Cronbach’s α value, which measures the power of correlation between sEFW and the neonatal birth weight (α ≥ 0.9, excellent correlation; 0.7 ≤ α < 0.9, good correlation; 0.6 ≤ α < 0.7, accepted correlation; 0.5 ≤ α < 0.6, poor correlation; and α < 0.5, unacceptable correlation). 

## 3. Results

### 3.1. Demographics

Overall, 28,834 women delivered in our institution during the study period, of which 1064 had twin gestations. After consolidating the database, 190 women with twin gestations had sonographic fetal evaluations performed within 14 days of delivery and were thus eligible for our analysis.

The demographic and obstetrical characteristics of the cohort are shown in Table 1. The median maternal age was 31.34 (26.42–36.2) years. One hundred thirty-six pregnancies were dichorionic-diamniotic (71.57%) twins, 34 (17.8%) were monochorionic-diamniotic twins, and the remaining were monochorionic-monoamniotic (0.52%). Only 38 (20%) pregnancies were complicated by maternal diabetes. For the entire cohort, the median gestational age at ultrasound evaluation was 35.54 (28.29–39.14) weeks, and the median sEFW was 2452 (834–5187) grams. The median gestational age at delivery was 36.37 (29.29–39.14) weeks, with a median birth weight of 2397 (775–3750) g. The median time interval from ultrasound evaluation to delivery was 5 (0–14) days. The majority of women delivered within 7 days of the sonographic evaluation (130/190, 68%), and over a third (82/190, 43.15%) delivered within 3 days of the evaluation.

### 3.2. sEFW Evaluation

Although there was no statistically significant difference in the sEFW between the first and second twins, the second twin had a statistically significant lower birth weight of 2434 (900–3750) vs. 2351 (775–3610), grams, *p* = 0.028), as shown in Table 2.

Overall, sEFW overestimated the actual neonatal birth weight. Specifically, there was almost no difference between the sEFW and birth weight for the first twin but a large overestimation for the second twin. 

### 3.3. Accuracy Measures

For all twins, there was wide variation in the absolute systematic error (median 6.86%, range 0.03 to 74.09). The median absolute systematic error and the proportion of estimates within 10% were similar between the first and the second fetuses (*p* > 0.05 for both). The random error was 8.54% for the entire cohort. Unlike the systematic error, the random error was slightly lower for the first twin (8.39 vs. 8.71 %). 

Reliability analysis demonstrated a good correlation with a Cronbach’s α value of 0.896 for the entire cohort with a slightly better correlation for the second twin. The Cronbach’s α value of the second twin was 0.911, suggestive of an excellent correlation, and the Cronbach’s α value of the first twin was 0.883, suggestive of a good correlation. 

The Euclidean distance was calculated to be 12.21 for the first twin and 12.27 for the second twin. The lower distance for the first twin reflects a higher prediction of neonatal birth weight.

Accuracy measurements for SGA prediction for the first and second twins are demonstrated in Table 3. The sensitivity of SGA prediction by sEFW was low for the entire cohort and both the first and second twins (59.26%, 62.07% and 57.69%, respectively). The specificity was high, with the best results for the second twin (92.75%). The PPV was low in both groups but higher for the second twin (PPV first twin: 45%; PPV second twin: 75%). The NPV was high for the entire cohort and for both the first and second twins (NPV 89%, 92.67% and 85.33%, respectively).

## 4. Discussion

Ultrasonographic estimation of fetal weight is routinely used in the management of multiple pregnancies and affects clinical decision-making regarding timing and mode of delivery. In this study, we aimed to evaluate the accuracy of sEFW in twin gestation. Specifically, we endeavored to assess the differences in accuracy between the first and second twins. Additionally, in order to evaluate the clinical impact of sEFW accuracy, we investigated the prediction of SGA at birth for both twins. 

Our study has several findings: 1. Overall, for the entire cohort, there was a good correlation between sEFW and birth weight; 2. the sonographic weight estimation of the second twin overestimated the actual birth weight; 3. the prediction of SGA was similar for both twins, however, with low sensitivity. The specificity and PPV were higher for the second twin. 4. There was no difference in the accuracy of predicting SGA between the first and second twins. 

The accuracy of sonography in predicting fetal weight has been studied in numerous studies [18,19,20]. One of the most comprehensive studies published by Benacerraf et al. estimated that 74% of neonates born were within 10% of the sonographically predicted sEFW [19]. The results of our study were similar: 66% of the entire cohort had an accurate estimation of fetal weight with a margin of error of 10%. Moreover, similar to previous studies, it was suggested that a shorter interval between sEFWs provides a more accurate estimation of neonatal weight [21,22,23].

Our study confirmed the previous reports showing a good correlation between sEFW and birth weight [22,23] and no difference in sEFW and birth weight between the first and second twins [23]. Unlike our results, Danon et al. [10] suggested lower accuracy in the sEFW of the second twin compared to the first twin. This difference might be explained by the high incidence of non-vertex presentation in the second twin group, causing dolichocephaly and smaller-than-anticipated BPD measurements [24,25].

Our results matched previous studies showing a tendency to underestimate the weight of the first twin and overestimate the weight of the second twin [10,22].

For sEFW prediction of SGA, overall we found high accuracy for both twins, as shown in previous studies [10]. We found low sensitivity in the prediction of SGA with high specificity, similar to other studies [10,22,23]. Conflicting results have been shown regarding sensitivity. While Kaouther et al. have shown good sensitivity for SGA prediction, our study, along with others [10,22,23], has found low sensitivity for the prediction of SGA. 

Cognitive biases are unconscious mental shortcuts or patterns that can influence how people perceive, interpret, and make decisions about information. Although diagnostic errors arising from cognitive biases are well studied in the radiology field, there remains a lack of research in the obstetric ultrasound field [26]. Our study highlights the importance of acknowledging that cognitive biases exist in the sonographic estimation of fetal weight. 

The strength of our study relies on the selection of cases for sonographic evaluation within 14 days of delivery, with the majority performed up to 7 days before delivery. Additionally, sonographic evaluation was undertaken by highly experienced ultrasound technicians or physicians who were ultrasound specialists. 

Our study is not free of limitations. First, this study is limited by its retrospective design. For this reason, no data was available regarding patients’ body mass index, demographics, or ethnic origin. Additionally, fetal data regarding gender was unavailable, which potentially could have affected the sonographic weight estimation prior to delivery and should have been evaluated as a confounding variable. Secondly, our study included a relatively small sample size of twins at all gestational ages, which could have affected our results. The inclusion of preterm deliveries that are potentially related to placental insufficiency complications during pregnancy may have influenced the proportion of SGA or growth-restricted fetuses. Therefore, future studies are needed to further study and validate our findings. Thirdly, chorionicity was evaluated sonographically without validation using postpartum placental examinations. Lastly, although care was taken to correctly name the first and second twins, we could not retrospectively validate that the presenting twin in ultrasound was always the first delivered, particularly in cases of cesarean section. 

## 5. Conclusions

In conclusion, twin gestations are prone to growth abnormalities, and fetal weights are typically smaller at term than in singleton pregnancies. Our study shows that sEFW has no difference in predicting birth weight for first and second twins, with high accuracy in predicting SGA but low sensitivity. 

## Figures and Tables

**Table 1:** Study cohort.

	Entire Cohort
Maternal age, years	31 (18–44)
Maternal diabetes (DM and GDM)	38 (20%)
Preeclampsia	22 (11.6%)
Gestational age at sEFW, weeks	35.57 (28.29–39.14)
Gestational age at delivery, weeks	36.86 (29.29–39.14)
Ultrasound-to-delivery interval	
■ Three-day interval	164/380, 43.15%
■ Seven-day intervall	260/380, 68.42%
Chorionicity	BCBA 136/190 (71.57) MCBA 34/190 (17.89) MCMA 1/190 (0.52)

Numbers are presented as median (range) for continuous variables and N (%) for categorical ones. sEFW–sonographically estimated fetal weight; DM—diabetes mellitus; GDM—gestational diabetes.

**Table 2:** Accuracy measures for first and second twins.

	First Twin	Second Twin	Entire Cohort	*p* Value
sEFW, grams	2436 (878–5187)	2458 (834–3504)	2452 (834–5187)	0.850
BW, grams	2434 (900–3750)	2351 (775–3610)	2397 (775–3750)	**0.028**
Systematic error	−0.86 (−37.94–74.09)	3.03 (−29.12–67.68)	0.97 (−37.94–74.09)	**0.001**
Absolute systematic error	7.08 (0.03–74.09)	6.61 (0.03–67.68)	6.86 (0.03–74.09)	0.450
Random error	8.39	8.71	8.54	
Proportion of estimation < 10%	124/190 (65.3)	129/190 (67.9)	253/380 (66.6)	0.587
SGA by sEFW	40/190 (21.1)	40/190 (21.1)	80/380 (21.1)	1
SGA by BW	29/190 (15.3)	52/190 (27.4)	81/380 (21.3)	**0.004**
EFW > BW	86/190 (45.3)	119/190 (62.6)	205/380 (53.9)	**<0.001**
Reliability analysis	0.883	0.911	0.896	

Numbers are presented as median (range). Significant differences are presented in bold (*p* < 0.005); sEFW–sonographically estimated fetal weight; BW–birth weight; SGA–small for gestational age.

**Table 3:** Accuracy measures for SGA prediction.

	First Twin	Second Twin	Entire Cohort
Sensitivity (%)	62.07%	57.69%	59.26%
Specificity (%)	86.34%	92.75%	89.30%
PPV (%)	45%	75%	60%
NPV (%)	92.67%	85.33%	89.00%
+LR	4.54 (2.81–7.35)	7.96 (4.2–15.11)	5.54 (3.81–8.05)
−LR	0.44 (0.27–0.70)	0.46 (0.33–0.63)	0.46 (0.35–0.6)
Accuracy	82.63%	83.16%	82.89%

PPV = positive predictive value; NPV = negative predictive value; +LR = positive likelihood ratio; −LR = negative likelihood ratio.

## Data Availability

The data presented in this study are available on request from the corresponding author.
